# Chinese medicines in the treatment of experimental diabetic nephropathy

**DOI:** 10.1186/s13020-016-0075-z

**Published:** 2016-02-24

**Authors:** Jing-Yi Liu, Xiao-Xin Chen, Sydney Chi-Wai Tang, Stephen Cho-Wing Sze, Yi-Bin Feng, Kai-Fai Lee, Kalin Yan-Bo Zhang

**Affiliations:** School of Chinese Medicine, Li Ka Shing Faculty of Medicine, The University of Hong Kong, 10 Sassoon Road, Hong Kong, People’s Republic of China; Department of Medicine, Li Ka Shing Faculty of Medicine, The University of Hong Kong, 10 Sassoon Road, Hong Kong, People’s Republic of China; Obstetrics and Gynaecology, Li Ka Shing Faculty of Medicine, The University of Hong Kong, 10 Sassoon Road, Hong Kong, People’s Republic of China

## Abstract

Diabetic nephropathy (DN) is a severe micro vascular complication accompanying diabetes mellitus that affects millions of people worldwide. End-stage renal disease occurs in nearly half of all DN patients, resulting in large medical costs and lost productivity. The course of DN progression is complicated, and effective and safe therapeutic strategies are desired. While the complex nature of DN renders medicines with a single therapeutic target less efficacious, Chinese medicine, with its holistic view targeting the whole system of the patient, has exhibited efficacy for DN management. This review aims to describe the experimental evidence for Chinese medicines in DN management, with an emphasis on the underlying mechanisms, and to discuss the combined use of herbs and drugs in DN treatment.

## Background

Diabetic nephropathy (DN) is a serious micro vascular complication in patients with diabetes mellitus (DM), affecting approximately 40 % of patients with type 1 or type 2 DM [1, 2]. It is the predominant cause of chronic kidney disease and renal failure, and is closely associated with many micro vascular diseases, leading to financial and medicinal burdens [3]. Continued hyperglycemia associated with DM is the major cause of kidney dysfunction with metabolic and hemodynamic disorders arising from oxidative stress and inflammation [4].

During DN progression, progressive alterations developfrom hyperfiltration through micro albuminuria to macro albuminuria, and finally to renal failure [5]. Renal structural changes are found in the nephrons, especially in the primary part of the glomerulus, including podocyte loss, glomerular basement membrane (GBM) thickening, endothelial cell dysfunction, and mesangial extracellular matrix (ECM) expansion, resulting in protein leakage into the urine [6]. Pulmonary dysfunction [7], hyperlipidemia and non-alcoholic fatty liver disease [8], cardiovascular disease [9], and even heart failure [10] have been reported to be positively associated with DN progression. Therefore, synergistic therapies targeting multiple mediators of DN are required for effective therapeutic strategies [4].

**Fig. 1:**
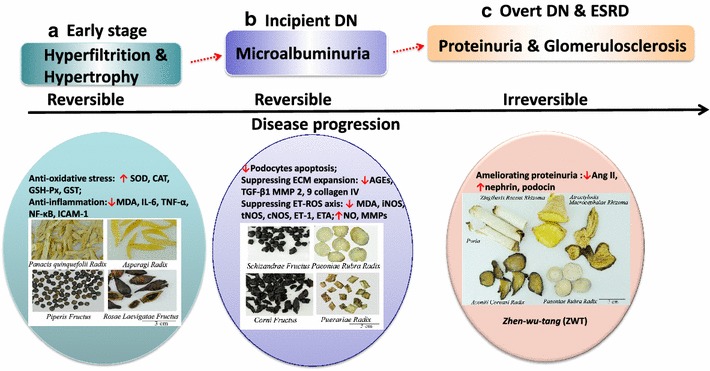
Natural course of diabetic nephropathy (DN) progression and Chinese medicine (CM) interventions in different stages. **a** In the early stage characterized by hyperfiltration and hypertrophy, CMs have therapeutic effects based on their anti-oxidant or anti-inflammatory activities. Representatives are *Panax quinquefolium*, *Asparagus racemosus*, *Rosa laevigata*, and *Piper auritum* [5, 42–44]. **b** In the incipient DN stage characterized by microalbuminuria, CMs such as *Cornus officinalis*, *Abelmoschus manihot*, *Schisandrae chinensis*, and *Paeonia lactiflora* exhibit anti-microalbuminuric effects and may slow down the propagation of DN [19, 21, 46, 47]. The mechanisms involve protecting podocytes, and suppressing extracellular matrix (ECM) expansion and the endothelin-reactive oxidative species (ET-ROS) axis. As both the early and incipient stages of DN are at least partially reversible, CM interventions, which have superior effects based on their anti-oxidant, anti-inflammatory, and other renoprotective activities, are recommended as early as possible. **c** In the overt and end-stage renal disease (ESRD) stages of DN characterized by proteinuria and glomerulosclerosis, respectively, CM prescriptions, such as *Zhen*-*wu*-*tang* (ZWT; also called Shinbu-to in Japan) consisting of five herbs including Common Monkshood root, Poria, White Peony root, *Atractylodis rhizome*, and *Zingiberis rhizome*, have demonstrated optimal effects on ameliorating proteinuria by suppressing the hyperactivity of the renal renin–angiotensin system [72]

The experimental models used for studying Chinese medicines (CMs) in DN treatment are diverse. For in vivo studies, different doses of streptozotocin (STZ) are administered to mimic type 1 or type 2 DM. Examples of the CMs that have been investigated are *Glycyrrhizauralensis* (*gan*-*cao*), *Carumcarvi* (*zang*-*hui*-*xiang*), *Allium sativum* (*da*-*suan*), and *Mesonaprocumbens* (*xian*-*cao*) [11–14]. In addition, alloxan (ALX)-induced mice, db/db mice, KK-Ay mice, and Otsuka Long-Evans Tokushima Fatty (OLETF) rats have been reported for investigation of CMs in DN treatment [15–18]. Meanwhile, glomerular endothelial cells, mouse podocyte cells, renal proximal epithelial cells, murine hepatocytes, mouse mesangial cells, and human mesangial cells are used as in vitro models for anti-DN mechanism studies [19–27]. By applying these models, the majority of studies have reported that CMs such as *Acacia nilotica* pods (*jin*-*he*-*huan*) [28], *Artemisia campestris* (*huang*-*ye*-*hao*) [29], *Paeonialactiflora* (*shao*-*yao*) [30], and *Schisandra chinensis* (*wu*-*wei*-*zi*) [21, 31] exhibited beneficial effects on all stages of experimental DN and may protect multiple organs. Grapevine leaf (*Vitis labrusca*) extract was reported to exert hepatoprotective, cardioprotective, and renoprotective effects [32]. Moreover, CM preparations such as *Fufang Xueshuantong Capsule* (*fu*-*fang*-*xue*-*shuan*-*tong*-*jiao*-*nang*), *Zhengqing Recipe* (*zheng*-*qing*-*fang*), and *Danggui Buxue Tang* demonstrated benefits for DN patients [33–35]. Representative CMs for the treatment of DN at different stages of disease progression and their underlying mechanisms are shown in Fig. 1.

This article aims to review the experimental evidence for the effectiveness of CMs in DN management, with emphasis on their underlying mechanisms, and to discuss the combined use of CM herbs and chemical drugs in DN treatment.

### Search strategy and selection criteria

We searched for the terms “traditional Chinese medicine”, “holistic therapy”, and “traditional Chinese medicine prescriptions (or formula)” in combination with “diabetic nephropathy” and “diabetes” in PubMed, Google Scholar, and Web of Science between 1990 and 2014. Manual searches of in-text references from the selected articles were further performed. Studies were included if in vivo models were used to investigate the nephroprotective effects and mechanisms of CMs. Unpublished reports, Letters to the Editor, and the studies that only used in vitro models or did not provide information about the duration of animal studies were excluded.

### CMs in experimental DN management

#### CMs intervention in the early stage of experimental DN

**Fig. 2:**
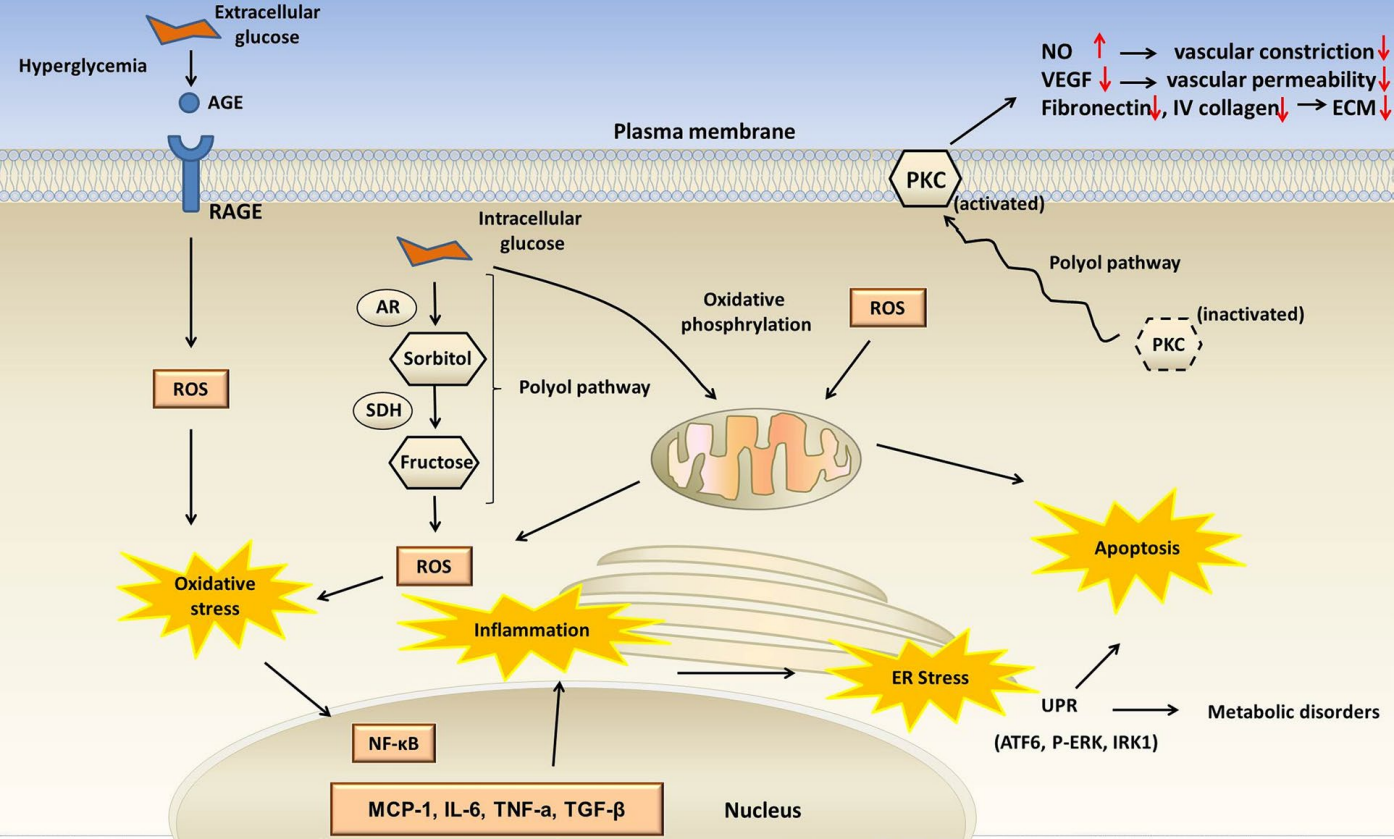
Potential signaling pathways involved in diabetic nephropathy (DN) pathogenesis. Activation of receptor of advanced glycation end-products (RAGE) by advanced glycation end-products (AGEs) results in reactive oxidative species (ROS) overproduction, leading to oxidative stress. Meanwhile, the polyol pathway activated by intracellular glucose further aggravates the oxidative stress. Activation of protein kinase C (PKC) via the polyol pathway is another major source of ROS production. Mitochondrial damage also contributes to ROS production. ROS overproduction and impaired anti-oxidant response cause oxidative stress, which activates nuclear factor (NF)-κB and upregulates monocyte chemoattractant protein (MCP)-1, interleukin (IL)-6, tumor necrosis factor (TNF)-α, and transforming growth factor (TGF)-β. Thereafter, the inflammation induces endoplasmic reticulum (ER) stress via unfolded protein response pathways, resulting in metabolic disorders and apoptosis

The potential signaling pathways involved in DN pathogenesis regulated by CMs are shown in Fig. 2. The early stage of DN is characterized by hyperfunction and hypertrophy arising from oxidative stress and inflammation [3, 36, 37]. Under chronic hyperglycemia, the extracellular glucose forms advanced glycation end-products (AGEs). Activation of receptor of advanced glycation end-products (RAGE) on the plasma membrane has been proposed to contribute predominantly to the overproduction of reactive oxidative species (ROS) [38]. Meanwhile, the polyol pathway of glucose metabolism activated by the intracellular glucose further aggravates the oxidative stress. Other major sources of excess ROS were reported to be enhanced protein kinase C (PKC) activity caused by activation of the polyol pathway [39] and mitochondrial ROS production in response to mitochondrial damage. As a consequence, nuclear factor (NF)-κB becomes activated, followed by stimulation of pro-inflammatory cytokines (e.g., interleukin [IL]-6), chemokines (e.g., monocyte chemoattractant protein [MCP]-1), adhesion molecules (e.g., intercellular adhesion molecule 1 [ICAM1], vascular cell adhesion protein 1 [VCAM1]), and nuclear receptors (e.g., peroxisome proliferator-activated receptor [PPARs]) [40]. Thereafter, the inflammation induces endoplasmic reticulum (ER) stress via unfolded protein response pathways, resulting in metabolic disorders and apoptosis. Besides, subsequent macrophage infiltration into renal tissues leads to prolonged micro inflammation, thus aggravating the progression of DN. Numerous CMs are applied at this point to control this reversible stage of DN [41]. *Asparagus racemosus* (*lu*-*sun*), *Radix Astragali* (*huang*-*qi*), *Rosa laevigata* (*jin*-*ying*-*zi*), and *Piper auritum* (*hu*-*jiao*) were reported to enhance the activities of superoxide dismutase (SOD) and glutathione peroxidase (GSH-Px), leading to attenuation of the oxidative stress [5, 42–44].

#### CMs intervention in the incipient stage of experimental DN

The development of micro albuminuria was reported as an indicator of the incipient stage of DN, arising from endothelial dysfunction [38, 45]. Renal hypertrophy and hyperfiltration induced functional and structural alterations, resulting in micro albuminuria and hypertension, leading to glomerulus sclerosis, and progressing to incipient DN. *Cornus officinalis* (*shan*-*zhu*-*yu*), *Abelmoschus manihot* (*huang*-*shu*-*kui*), *Schisandrae chinensis* (*wu*-*wei*-*zi*), *and Paeonia lactiflora* (*shao*-*yao*) were reported to exhibit anti-micro albuminuria effects, thereby slowing down DN progression [19, 21, 46, 47].

#### CMs intervention in the overt and end-stage renal disease (ESRD) stages of experimental DN

After the incipient stage of DN and under hyperglycemic conditions, mesangial nodules and tubule interstitial fibrosis develop, leading to proteinuria and nephrotic syndrome, and eventually to the overt stage of DN, which is characterized by persistent proteinuria [6]. Without effective control, patients in this stage will deteriorate to ESRD with uremia. As the kidney disease progresses, physical changes in the kidneys often lead to increased blood pressure and cardiovascular disease. In this stage, angiotensin-converting enzyme (ACE) inhibition is the conventional intervention [48]. The goal of treatment is to prevent the progression from micro albuminuria to macro albuminuria, and multiple and more intensive strategies are strongly advised. Avosentan was reported to reduce albuminuria in patients with type 2 DM and overt nephropathy by inhibiting ACE and blocking angiotensin receptors, but can also induce significant fluid overload and congestive heart failure [49]. *Averrhoa carambola* L. (*yang*-*tao*), *Salvia miltiorrhiza* (*dan*-*shen*), and *Picrorrhiza Rhizoma* (*hu*-*huang*-*lian*) can ameliorate DN symptoms safely [50–52]. Representative CMs and their related mechanisms are summarized in Table 1.

**Table 1:** Chinese medicines used in the management of experimental diabetic nephropathy

Species	Medicinal part	Extract/Compound	DN model	Nephro-protective Mechanisms	Pharmacodynamic indicators	Duration	Ref.
*Eclipta alba* (*han*-*lian*-*cao*)	–	Ethanol extract	STZ rat	↓α-glucosidase and aldose reductase activities	FBG, HbA1C, urea, uric acid, UCr, insulin	3 weeks	[76]
*Gymnemamontanum*Hook (*shi*-*geng*-*teng*)	–	Ethanol extract	ALX rat	↓TBARS, hydroperoxides; ↑SOD, CAT, GSH-Px, GST	FBG, insulin, urea, Cr, uric acid	3 weeks	[77]
*Cinnamomumzeylanicum* (*xi*-*lan*-*rou*-*gui*)	–	Aqueous extract	STZ rat	↑UCP-1, GLUT4	FBG, K/B ratio, insulin, HDL, TC, TG, Cr, histopathology	22 days	[78]
*Panaxnotoginseng* (*san*-*qi*)	Roots	Notoginoside	STZ rat	↓VEGF; ↑BMP-7	Cr, CCr, Ualb	4 weeks	[79]
*Mesonaprocumbens*Hemsl (*xiancao*)	–	Aqueous extract	STZ rat	↓TSP-1	Body weight, FBG, histopathology	4 weeks	[14]
*Piper auritum* (*hu*-*jiao*)	Leaves	Hexane extract	STZ rat	↓AGEs, serum glycosylated protein, LDL glycation, glycation hemoglobin, renal glucose, thiobarbituric acid-reactive substance; ↑SOD, CAT, GPx and GSH	Kidney oxidative stress	4 weeks	[44]
*Smallanthussonchifolius* (*xue*-*lian*)	Leaves	Aqueous extract	STZ rat	↓TGF-β1, Smad2/3, collagen III, collagen IV, laminin-1, FN	FBG, insulin, UAE, Cr, kidney hypertrophy, GBM thickening	4 weeks	[80]
*Milk thistle* (*nai*-*ji*-*cao*)	–	Silymarin	STZ rat	↓Lipid peroxidation; ↑CAT, SOD, GPx	FBG, serum urea, Cr, Ualb	4 weeks	[81]
–	–	Curcumin	STZ rat	↓eNOS, ET-1, TGF-β1, FN, NF-κB, p300	ECM	4 weeks	[82]
*Allium sativum*L. (*da*-*suan*)	–	–	STZ rat	↓TBARS; ↑GSH	FBG, insulin, TG, TC, CCr, UAE, NAG	30 days	[13]
*Psidiumguajava*L. (*fan*-*shi*-*liu*)	Leaves	Total triterpenoids	HFD + STZ rat	↓Hyperglycemia	FBG, insulin, Cr, BUN, capillary, base-membrane incrassation, glomerular swelling, cysts and tubules edema	6 weeks	[83]
*Panaxnotoginseng* (*san*-*qi*)	Roots	Notoginoside	STZ rat	↓TGF-β1; ↑Smad7	FBG, renal index, CCr, UAlb	6 weeks	[84]
*Trigonellafoenumgraecum*(*xiang*-*cao*)	Seeds	Aqueous extract	HFD + STZ rat	↓MDA, 8-hydroxy-2′-deoxyguanosine, renal cortex DNA; ↑SOD, CAT	FBG, K/B ratio, Cr, BUN, UAlb, and CCr, GBM	6 weeks	[85]
*Schisandraechinensis* (*wu*-*wei*-*zi*)	Fruits	Ethanol extract	STZ mice	↓EMT, α-SMA, PAI-1, E-cadherin, Snail; ↑E-cadherin, α-SMA	ACR, UAE, ECM deposition, podocyte loss and integrity of the slit diaphragm	7 weeks	[21]
–	–	Curcumin	STZ mice	↓COX-2, caspase-3, F- to G-actin cleavage; ↑p38-MAPK, HSP25	UAlb, ACR	7 weeks	[24]
*Panax ginseng* (*ren*-*shen*)	–	ginsenoside 20(S)-Rg(3)	OLETF rats	↓TBARS, iNOS, CML	FBG, CCr, UAE, urine volume	50 days	[18]
*Polygonummultiflorum*Thunb (*he*-*shou*-*wu*)	–	Tetrahydroxystilbene	STZ rat	↓TGF-β1, COX-2; ↑CAT, SOD, GSH-Px, SIRT1	TC, TG, BUN, Cr, UAlb, K/B ratio, MDA	8 weeks	[25]
*Paeonialactiflora*Pall. (*shao*-*yao*)	–	Total glucosides	STZ rat	↓Macrophages accumulation and proliferation; ↑p-JAK2, p-STAT3	UAlb	8 weeks	[47]
*Aceranthussagittatus* (*yin*-*yang*-*huo*)	–	Icariin	STZ rat	↓MDA, Hyp, TGF-β1, collagen IV; ↑SOD	FBG, Cr, BUN, histopathology	8 weeks	[86]
*Angelica acutiloba* (*dang*-*gui*)	Roots	Aqueous ethanol extract	STZ rat	↓NF-κB, TGF-β1, FN, AGEs, RAGE	FBG, UAlb, UAE, CCr, ECM expansion	8 weeks	[87]
*Salvia miltiorrhiza* (*dan*-*shen*)	–	Aqueous extract	STZ rat	↓TGF-β1, AGEs, RAGE, collagen IV and ED-1	FBG, UAlb, UAE	8 weeks	[51]
*Tripterygium wilfordii* (*lei*-*gong*-*teng*)	–	Multi-glycoside	STZ rat	↓Mesangial cell proliferation, α-SMA, collagen 1	Body weight, UAlb, FBG, Cr, BUN, histopathology	8 weeks	[88]
*Hibiscus sabdariffa* L (*luo*-*shen*-*hua*)	Flowers	Polyphenols	STZ rat	↓TBARS; ↑CAT and GSH	K/B ratio, proximal convoluted tubules, TG, TC, LDL	8 weeks	[89]
*Panaxquinquefolium* (*xi*-*yang*-*shen*)	Roots	Ethanol extract	STZ+ db/db mice	↓Oxidative stress, NF-κB p65, ECM, vasoactive factors	Albuminuria and mesangial expansion	6 and 8 weeks	[90]
*Rheum officinale* (*da*-*huang*)	–	Rhein	db/db mice	↓TGF-β1, FN	UAE, ECM, TC, TG, LDL-C, Apo E	8 weeks	[91]
*Averrhoa carambola* L (*yang*-*tao*)	Roots	2-dodecyl-6-methoxycyclohexa-2,5-diene-1,4-dione	KKAy mice	↓Hyperglycemia, AGE, NF-κB, TGF-β1, CML; ↑SOD and GSH-Px activities	Proteinuria, Cr, CCr, serum urea-N, ECM expansion	8 weeks	[17]
*Radix Astragali* (*huang*-*qi*)	Roots	Aqueous extract	STZ rat	↓MDA, IL-6, TNF-α, NF-κB, PKCα; ↑SOD and GSH-Px activities	FBG, body weight, Cr	60 days	[42]
*Glycyrrhizauralensis* (*gan*-*cao*)	–	–	STZ rat	↓MDA; ↑GSH, SOD and CAT	FBG, body weight, histopathology	60 days	[11]
*Acacia nilotica* (*jin*-*he*-*huan*)	Pods	Aqueous methanol extract	STZ rat	↓Hyperglycemia, LPO, ↑SOD and GSH activities	FBG, serum urea, Cr, histopathology	60 days	[28]
*Portulacaoleracea* (*ma*-*chi*-*xian*)	–	Aqueous extract	db/db mice	↓TGF-β1, AGEs, ICAM-1, NF-κB p65	FBG, Cr, water intake and urine volume	10 weeks	[92]
–	–	Genistein	STZ mice	↓ICAM-1, gp91 and TBARs; ↑ phospho-tyrosine and phospho-ERK/ERK ratio	FBG, insulin, total protein, UAlb, urinary MCP-1 excretion	10 weeks	[93]
*Smilax glabra*Roxb (*tu*-*fu*-*ling*)	Rhizome	Astilbin	STZ rat	↓TGF-β1, CTGF	Body weight, survival time, FBS	6 and 12 weeks	[94]
*Psidiumguajava*L. (*fan*-*shi*-*liu*)	Fruits	Aqueous + methanol extract	STZ mice	↓AR activity, ROS, IL-6, TNF-α, IL-1β, CML, MDA, AR and AGEs; ↑GSH, CAT, GSH-Px	Body weight, insulin	12 weeks	[95]
–	–	Caffeic acid, ellagic acid	STZ mice	↓Sorbitol dehydrogenase, AR, IL-1, IL-6, TNF-α, MCP-1	Body weight, urine volume, insulin, FBG, BUN, CCr, HbA1c, UAlb	12 weeks	[96]
*Trigonellafoenumgraecum*L. (*hu*-*lu*-*ba*)	Seeds	Seed powder	ALX rat	↓Glucose, urea, creatinine, sodium, potassium and IL-6 in serum, MDA and IL-6 in kidney; ↑SOD and CAT activities, GSH	Glomerular mesangial expansion	12 weeks	[97]
*Cornus officinalis* (*shan*-*zhu*-*yu*)	Fruits	–	HFD + STZ rat	↓FBG, NAG, mALB; ↑insulin and Wilms tumor 1 in glomeruli	FBG, mALB, UCr, BUN, NAG, histopathology	12 weeks	[19]
*Euonymus alatus* (*wei*-*mao*)	Leaves and branches	Aqueous extract	Uninephrectomy + STZ rat	↓TGF-β1	Blood lipids, UAlb, HbA1c, ECM expansion and glomerulus sclerosis	12 weeks	[98]
*Aster koraiensis* (*zi*-*yuan*)	Aerial part	Ethanol extract	STZ rat	↓AGEs accumulation, Bax; ↑Bcl-2	FBG, HbA1c, UAE, histopathology	13 weeks	[99]
*Rosa laevigata*Michx. (*jin*-*ying*-*zi*)	Fruits	Aqueous extract	STZ rat	↓MDA, ROS, NF-κB p65, MCP-1;↑SOD and antioxidant activities, IκBα	Kidney oxidative stress	24 weeks	[43]
*Abelmoschusmanihot*L. (*huang*-*shu*-*kui*)	Flowers	Total flavone glycosides, hyperoside	STZ rat	↓Glomerular cell and podocytes apoptosis, caspase-3, caspase-8	ACR, UAlb	24 weeks	[46]

*AGEs* advanced glycation end products, *ALX* alloxan, *AR* aldose reductase, *ACR* urinary microalbumin to creatinine ratio, *BMP* bone morphogenetic protein, *BUN* blood urea nitrogen, *CAT* catalase, *CCr* creatinine clearance rate, *CML* N(epsilon)-(carboxymethyl) lysine, *CTGF* connective tissue growth factor, *COX* cyclooxygenase, *ECM* extracellular matrix, *ED-1* monocyte/macrophage, *ET-1* endothelin-1, *EMT* epithelial-to-mesenchymal transition, *ERK* extracellular signal-regulated kinases, *FBG* fasting blood glucose, *FN* fibronectin, *GBM* glomerular basement membrane, *GLUT* glucose transporter, *GSH-Px* glutathione peroxidase, *GST* glutathione-S-transferase, *HFD* high fat diet, *HDL* high density lipoprotein, *HSP* heat shock protein, *Hyp* hydroxyproline, *ICAM* intercellular adhesion molecule, *JAK* janus kinase, *K/B* kidney/body weight, *LDL* low density lipoprotein, *LPO* lipid peroxidation, *iNOS* inducible nitric oxide synthase, *eNOS* endothelial nitric oxide synthase, *NAG* N-acetyl-beta-D-glucosaminidase, *NF-κB* nuclear factor κB, *MAPK* mitogen-activated protein kinase, *mALB* microalbuminuria, *MCP* monocyte chemotactic protein, *MDA* malondialdehyde, *PAI* plasminogen activator inhibitor, *ROS* reactive oxidative species, *RAGE* receptor of advanced glycation end-products, *STAT3* signal transducer and activator of transcription 3, *α-SMA* α-smooth muscle actin, *STZ* Streptozotocin, *SIRT1* Sirtuin 1, *SOD* superoxide dismutase, *TARS* thiobarbituric acid reactive substances, *TGF* transforming growth factor, *TG* triglyceride, *TC* total cholesterol, *TSP-1* thrombospondin-1, *UAlb* urinary microalbumin, *UAE* urinary albumin excretion, *UCr* urinary creatinine, *UCP* uncoupling protein, *VEGF* vascular endothelial growth factor

**Fig. 3:**
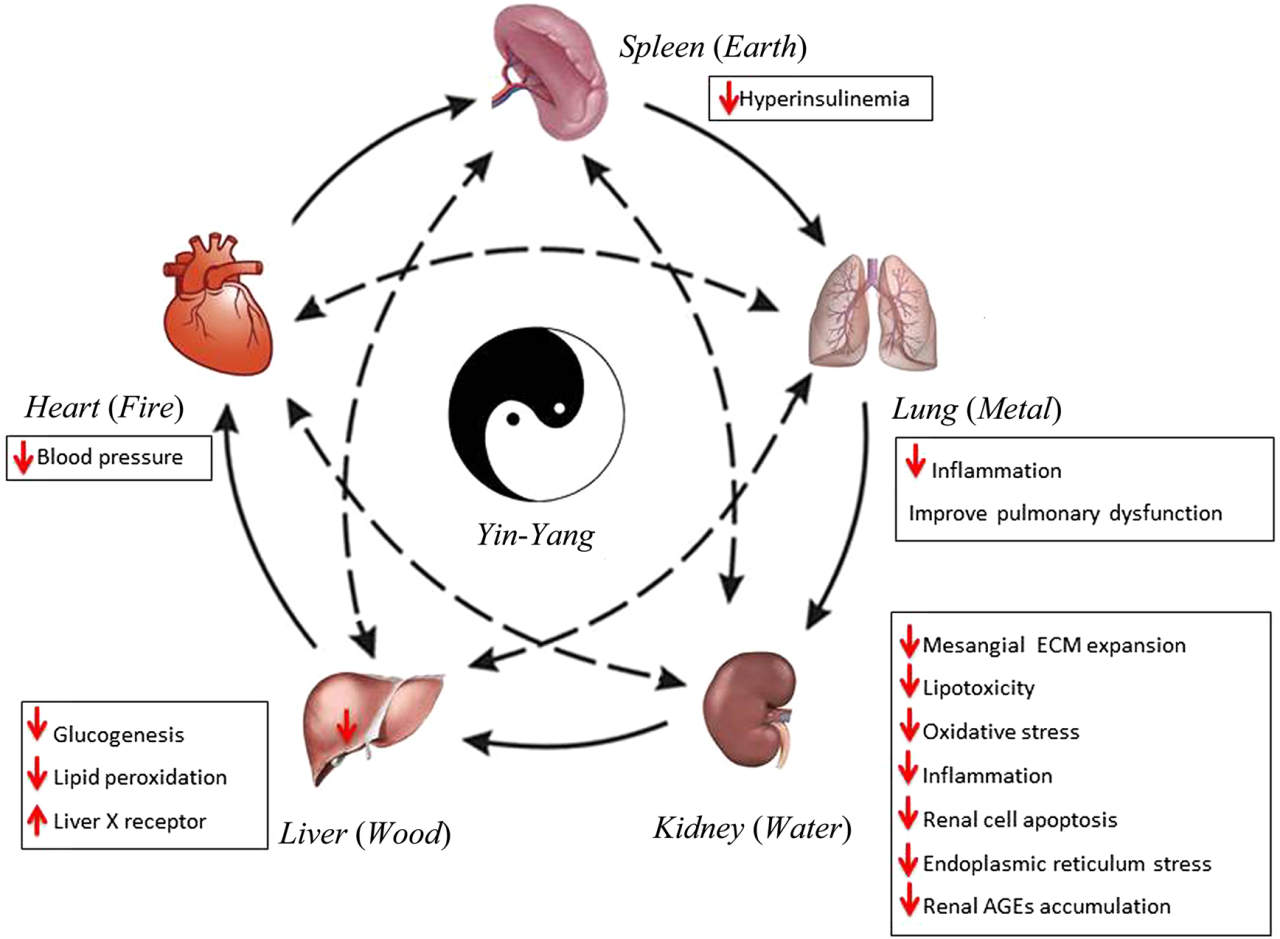
Schematic diagram integrating the Chinese medicine (CM) view on the holistic therapy and modern pathogenesis concepts of diabetic nephropathy (DN). The core shows the holistic view of DN under CM theory, which is based on the *Yin*-*Yang* and *Five Elements* theories. The regular functioning of the human body relies on the coordination of *Yin* and *Yang* in a unity of opposites, and the liver, heart, spleen, lung, and kidneys are respectively related to *wood*, *fire*, *earth*, *metal*, and *water* [53]. In particular, the spleen in CM is a functional organ that governs transport and transformation in a close relationship with the stomach and pancreas [73–75]. This theory reflects the unification and integration together with the impact caused by the breakdown of the balance as a consequence of overacting and counteracting relationships, which is of practical significance in CM clinical practice. The peripheral annotations imply recent therapeutic strategies against DN specific to individual organs. The *solid arrows* denote interpromoting relationships. The *dashed arrows* indicate interacting/counteracting relationships

Besides targeting the specific molecules involved in DN pathogenesis to exert anti-hyperglycemic and nephroprotective effects, CM has unique characteristics in DN management. In CM, DN is not only a kidney disease, but also an embodiment of the systemic disease in the kidney, which is in accordance with the latest findings for DN pathogenesis [7, 8, 38]. The pathogenesis of DN may be closely related to the dysfunction or impairment of other organs, and therefore treatments for diseases in other organs may be helpful for the amelioration of DN, especially in the overt and ESRD stages. The normal functioning of the human body relies on the coordination of *yin*and *yang*, and the five *zang* organs (*wuzang*), i.e., the *liver* (*gan*), *heart* (*xin*), *spleen* (*pi*), *lung* (*fei*), and *kidney* (*shen*), are respectively related to *wood* (*mu*), *fire* (*huo*), *earth* (*tu*), *metal* (*jin*), and *water* (*shui*) and connected under the laws of inter promotion and interaction (Fig. 3) [53]. Once a significant imbalance occurs, certain symptoms of the kidneys inevitably and predictably arise.

Under hyperglycemic conditions, the oxidative stress and inflammation affect the blood circulatory system, consequently leading to the dysfunction of multiple organs. Cardiovascular disease causes even more deaths than ESRD in patients with DN [38]. The degree of pulmonary function impairment was found to be positively associated with the stage of DN progression [7]. Besides, liver X receptor (LXR) agonists, which are commonly used to treat hyperlipidemia and non-alcoholic fatty liver disease, were shown to ameliorate DN by inhibiting the expressions of osteopontin and other inflammatory mediators in the kidney cortex [8]. Moreover, during DN pathogenesis, glomerular hypertrophy was found to be associated with hyperinsulinemia [54], and has been proposed as a novel therapeutic target for DN [55]. As a systematic micro vascular thrombosis combined with metabolic disorders, DN influences the whole internal environment, and its pathogenesis may be closely related to the dysfunction of other organs.

From this perspective, CM as a therapeutic approach targeting multiple organs is preferred to improve the overall health of DN patients. Experimentally, grapevine (*Vitis labrusca* L.) leaves exhibited hepatoprotective, cardioprotective, and renoprotective effects in Wistar rats [32]. Besides, extracts from *S. miltiorrhiza* exhibited a regulatory effect on the expression of LXR-α in hyperlipidemic rats [56]. Furthermore, *Liuwei Dihuang* Decoction exhibited a protective effect on early DN in STZ rats [57]. Additionally, a CM prescription, *kangen*-*karyu*, exhibited hepatoprotective/renoprotective activities through the inhibition of AGE formation and fibrosis-related protein expressions in type 2 diabetes [58]. Yamabe and colleagues systematically conducted a series of experiments to investigate the anti-diabetic effects of a CM prescription, *hachimi*-*jio*-*ga*, and reported findings for the whole prescription and its constituents as well as for the bioactive compound [59–64]. Other selected CM prescriptions for DN treatments and their respective molecular mechanisms are shown in Table 2. In particular, single herbs (e.g., *Auricularia auricula*, *hei*-*mu*-*er*) and CM prescriptions (e.g., *Danggui Buxue Tang* and *Gui Qi Mixture*) produced better beneficial effects than conventional anti-DN drugs by regulating blood lipid metabolism and lipoprotein lipase activity through the regulation of blood glucose based on their complex compound matrices [65–67]. The changes in blood glucose, triglyceride (TG), total cholesterol (TC), and high-density lipoprotein (HDL) were reversed by *Gui QiMixture*, but not by the ACE inhibitor benazepril in diabetic rats [68]. Similarly, the increases in fasting blood glucose (FBG), TG, and TC were attenuated, and the renal kidney/body weight (K/B) ratio, urinary albumin excretion (UAE), and creatinine clearance rate (CCr) in STZ-induced diabetic rats were ameliorated after 8 weeks of treatment with *Danggui Buxue Tang* compared with benazepril [69]. Collectively, CMs may exert synergetic effects targeting multiple organs, and benefiting the whole internal milieu of DN patients.

**Table 2:** Experimental studies on selected CM prescriptions in diabetes nephropathy management

CM preparations	DN model	Nephro-protective mechanisms	Pharmacodynamic indicators	Dosage	Duration	Ref.
*Xiao*-*chai*-*hu*-*tang*	STZ rat	↓TGF-β1, FN, and collagen IV,↑BMP-7, SOD	FBG, BUN, SCr, renal hypertrophy	200 mg/kg b.w	4 weeks	[100]
*LiuweiDihuang Decoction*	STZ rat	↓MDA, iNOS, tNOS, cNOS, ET-1, ET(A), ↑NO, MMP-2, MMP-9, GSH-Px, SOD	FBG, plasma insulin level	5, 10, or 15 g/kg b.w	4 weeks	[57]
*Tangshenling mixture plus benazepril*	STZ rat	↓ANF, GLUT1	UAE, CCr, K/B ratio	5 g/kg b.w	6 weeks	[71]
*DangguiBuxue Tang*	STZ rat	↓TGF-β1	K/B ratio, UAE, β(2)-MG concentrations, CCr, FBG, TC, TG	–	8 weeks	[69]
*Dang*-*gui and Huang*-*qi mixture*	STZ rat	↓TGF-β1, Ang II	FBG, TG, CHO, HDL, SCr, CCr, BUN, β(2)-MG,K/B ratio, GA	–	8 weeks	[68]
*Tangshenning Recipe*	STZ rat	↓TXB(2), TXB(2)/6-keto-PGF1 α, CGRP, MDA; ↑ET, SOD, GSH	–	35 g/kg b.w	8 weeks	[101]
*Shenbao Recipe*	STZ rat	↓CTGF, ↑MMP-9	UAlb, FBG, TC, SCr	13 g/kg b.w	8 weeks	[102]
*Wu*-*ling*-*san*	STZ rat	↓NF-κB, TGF-β1, FN, AGEs, mitochondrial TBARS, CML	UAE, UAlb, CCr, mesangial matrix expansion	2.5 g/kg b.w	10 weeks	[103]
*Zhen*-*wu*-*tang*	STZ rat	↓Ang II, ↑nephrin, podocin	Body weight, polyurea, UAE, SCr, BUN	320 mg/kg b.w.	12 weeks	[72]
*FufangXueshuantong Capsule*	HFD + STZ rat	↑GSH-px, SOD	UAE, CCr, masengial matrix expansion	450, 900, or 1800 mg/kg b.w	12 weeks	[104]
*Hachimi*-*jio*-*gan*	STZ rat	↓AGEs, sorbitol	FBG, UAE, CCr, serum glycosylated protein, BUN, serum albumin level, TG, TC	50,100, or 200 mg/kg b.w	15 weeks	[59]
*Kangen*-*karyu*	STZ mouse	↓AGEs, TGF-β1, collagen IV	FBG, BUN	100, 200 mg/kg b.w	18 weeks	[58]
*Hachimi*-*jio*-*gan*	OLETF rats	↓NF-κB, TGF-β1, FN, iNOS, cyclooxygenase-2, AGEs, TBARS	UAE, CCr, FBG	50, 100, or 200 mg/kg b.w	32 weeks	[61]
*Yiqiyangyinhuayutongluo recipe*	HFD + STZ rat	↑Nephrin	FBG, UAE, 24 h U-nephrin	0.8 g/kg b.w	32 weeks	[105]

*AGEs* advanced glycation end products, *ANF* atrial natriuretic factor, *Ang II* angiotensin II, *BMP* bone morphogenetic protein, *BUN* blood urea nitrogen, *CCr* creatinine clearance rate, *CHO* cholesterol, *CML* N(epsilon)-(carboxymethyl)lysine, *CGRP* calcitonin gene-related peptide, *CTGF* connective tissue growth factor, *ET* endothelin, *FBG* fasting blood glucose, *GA* glomerular area, *GLUT* glucose transporter, *TGF* transforming growth factor, *FN* fibronectin, *GSH-Px* glutathione peroxidase, *HDL* high density lipoprotein, *HFD* high fat diet, *K/B* kidney/body weight, *NF-κB* nuclear factor κB, *NO* nitric oxide, *cNOS* constitutive nitric oxide synthase, *eNOS* endothelial nitric oxide synthase, *iNOS* inducible nitric oxide synthase, *nNOS* constitutive nitric oxide synthase, *tNOS* total nitric oxide synthase, *MDA* malondialdehyde, *MMP* matrix metalloproteinase, *β (2)-MG* Urine β (2)-microglobin, *OLETF* otsuka long-Evans Tokushima Fatty, *PGF* prostaglandin F, *SCr* serum creatinine clearance rate, *STZ* streptozotocin, *SOD* superoxide dismutase, *TGF* transforming growth factor, *TG* triglyceride, *TC* total cholesterol, *TARS* thiobarbituric acid reactive substances, *TXB(2)* thromboxane B 2, *UAE* urinary albumin excretion rate, *UAlb* urinary microalbumin

At the ESRD stage, it is almost impossible to prevent the disease from becoming more severe, and dialysis may be the final resort for these patients. To provide a more cost-effective therapeutic approach, other potent remedies are urgently needed. In this regard, the combined use of herbs and drugs, and the development of new therapies are receiving increasing attention.

Modern drugs specifically aim to target disease-related molecules through definite pathways, whereas CM aims to exert synergetic effects and benefit the whole internal milieu of patients, leading to the possibility that the combined use of CMs and modern drugs may exert better therapeutic effects on diseases, especially for chronic and comprehensive DN. Currently, the combined use of herbs and drugs in the treatment of DN has been well-investigated. For example, the CM prescription *tangshenling* was combined with telmisartan to treat 80 patients with DN, and exhibited a better effect than telmisartan treatment alone [70]. Basic research corroborated that the *tangshenling* mixture had a synergetic effect with benazepril through a different signaling pathway, which involved down regulation of atrial natriuretic factor (ANF) in plasma and glucose transporter 1 (GLUT1) in the kidney when treating DN [71]. Herbs may reduce the permeability of the drug into the intestinal tract, and may also affect its metabolism in the liver and cause hypoglycemia. *Huang Kui* capsule reduced the absorption of glibenclamide and accelerated its metabolism. This herb–drug interaction deserves further research on the herb–drug pharmacokinetic interaction to enhance the therapeutic effects and avoid side effects.

## Limitations of this review

In many studies included in this review, the bioactivities of the CMs responsible for the anti-DN effects and their molecular targets were not identified. Phytochemical and molecular biological studies are needed to identify the bioactive constituents and to elucidate the underlying mechanisms. Moreover, this review only focused on studies using in vitro or in vivo DN models. Results from clinical trials investigating the use of CMs for the treatment of DN are needed to confirm the therapeutic effects of CMs in the future.

## Conclusion

CMs provides an alternative for DN management in all stages of experimental DN models, especially in the early and incipient stages of DN, and the synergistic administration of CM herbs with conventional drugs exhibited better efficacy than drugs alone in DN treatment.

## Abbreviations

ANF: atrial natriuretic factor
AGEs: advanced glycation end products
Ang II: angiotensin II
ALX: alloxan
AR: aldose reductase
ACE: angiotensin-converting enzyme
ARB: angiotensin receptor blocker
ACR: urinary microalbumin to creatinine ratio
BUN: blood urea nitrogen
BMP: bone morphogenetic protein
CAT: catalase
CCr: creatinine clearance rate
CGRP: calcitonin gene-related peptide
CHO: cholesterol
CTGF: connective tissue growth factor
CML: n(epsilon)-(carboxymethyl)lysine
DM: diabetes mellitus
DN: diabetic nephropathy
ER: endoplasmic reticulum
ET-1: endothelin-1
ESRD: end-stage renal disease
EMT: epithelial-to-mesenchymal transition
ECM: extracellular matrix
EMMPRIN: extracellular matrix metalloproteinase inducer
ERK: extracellular signal-regulated kinases
ED-1: monocyte/macrophage
FBG: fasting blood glucose
FN: fibronectin
GA: glomerular area
GFR: glomerular filtration rate
GMCs: glomerular mesangial cells
GBM: glomerular basement membrane
GSH-Px: glutathione peroxidase
GST: glutathione-S-transferase
GLUT: glucose transporter
HDL: high density lipoprotein
HFD: high fat diet
Hyp: hydroxyproline
iNOS: inducible nitric oxide synthase
ICAM: intercellular adhesion molecule
IGF: insulin-like growth factor
K/B: kidney/body weight
LPO: lipid peroxidation
LPL: lipoprotein lipase
LXR: liver X receptor
LDL: low density lipoprotein
NAG: n-acetyl-beta-D-glucosaminidase
eNOS: endothelial nitric oxide synthase
nNOS: constitutive nitric oxide synthase
tNOS: total nitric oxide synthase
MAPK: mitogen-Activated Protein Kinase
mALB: microalbuminuria
MDA: malondialdehyde
MMP: matrix metalloproteinase
MCP: monocyte chemotactic protein
OLETF: otsuka Long-Evans Tokushima Fatty
PPAR: peroxisome proliferator-activated receptor
PAI: plasminogen activator inhibitor
PK1: protein kinase 1
PGF: prostaglandin F
ROS: reactive oxidative species
RAGE: receptor of advanced glycation end-products
SGK: serum and glucocorticoid induced protein kinase
STZ: streptozotocin
SOD: superoxide dismutase
α-SMA: α-smooth muscle actin
SCr: serum creatinine clearance rate
TGF: transforming growth factor
CM: chinese medicine
TARS: thiobarbituric acid reactive substances
TIMP: tissue inhibitor of metalloproteinase
TG: triglyceride
TC: total cholesterol
TSP-1: thrombospondin-1
TXB(2): thromboxane B 2
UCr: urinary creatinine
β (2)-MG: urine β (2)-microglobin
UCP: uncoupling protein
UAlb: urinary microalbumin
UPR: unfolded protein response
UAE: urinary albumin excretion
VEGF: vascular endothelial growth factor
